# The multifaceted impact of the COVID-19 pandemic on colorectal cancer care: a systematic review of management disruptions, stage migration, and survival outcomes

**DOI:** 10.3389/fonc.2026.1828760

**Published:** 2026-08-19

**Authors:** Khaled Bonna, Abdulrahman Alshawaf, Faisal Abusharkh, Meshari Alenazi, Jamil Aljehany, Afaf Bazerbashi, Maein Mare Mohammed Alshaykhi, Dalah Ajam, Genin Hasanoğlu

**Affiliations:** 1 Department of Surgical Oncology, Oncology Center, College of Medicine, Mansoura University, Mansoura, Egypt; 2 Medical Department, College of Medicine, İstiniye University, Istanbul, Türkiye; 3 Reference Lab., Tabuk Cluster, Tabuk, Saudi Arabia; 4 College of Medicine, Taibah University, AL Madinah AL Munawwarah, Saudi Arabia; 5 Üsküdar University, Istanbul, Türkiye

**Keywords:** cancer care, colorectal cancer, COVID-19 pandemic, outcome, survival outcomes

## Abstract

**Background:**

The COVID-19 pandemic caused a catastrophic disruption to global healthcare systems. For colorectal cancer (CRC), the world’s third most prevalent cancer, the suspension of screening programs and reallocation of resources threatened to severely impact timely diagnosis and treatment, with pre-existing models predicting significant excess mortality from even short delays.

**Objective:**

This systematic review aimed to assess the impact of the COVID-19 pandemic on the management strategies, treatment outcomes, and survival rates of patients with colorectal cancer.

**Methods:**

A systematic review was conducted following PRISMA guidelines. A comprehensive search of PubMed was performed for studies published from 2020 onwards. Eligible studies included those focusing on adult CRC patients and reporting on changes in care pathways or outcomes due to the pandemic. Data on diagnostic delays, treatment modifications, stage at presentation, and survival were extracted and synthesized narratively.

**Results:**

The synthesis of results from the included studies revealed a cascade of negative consequences. There was a severe global reduction in CRC screening, with participation declining by 30-80% and colonoscopy volumes falling by up to 92%, leading to a pool of “missing” diagnoses. This resulted in a significant stage shift, with a higher proportion of patients presenting with advanced stage (e.g., Stage IV) and locally advanced tumors. Treatment protocols were modified, including reduced elective surgical volumes and increased use of neoadjuvant therapy. Consequently, patients diagnosed during the pandemic faced a significantly higher mortality risk, a trend exacerbated by COVID-19 co-infection, which was associated with dramatically worse survival. The pandemic also exacerbated health disparities and imposed a profound psychosocial burden on patients.

**Conclusion:**

The COVID-19 pandemic had a detrimental impact on every facet of colorectal cancer care, from screening and diagnosis to treatment and outcomes, leading to more advanced disease at presentation and worsening survival. These findings underscore the critical need to develop resilient cancer care systems that can maintain essential services during future public health crises.

**Systematic review registration:**

https://www.crd.york.ac.uk/PROSPERO/, identifier CRD42025638100.

## 1 Introduction

Coronaviruses are large RNA, enveloped viruses. They are known to cause conditions ranging from common colds in adults to gastroenteritis in infants. These viruses were given the name of corona because of the spikes on their surface giving them a crown-like shape. In the recent years they have become well known because of the pandemic that they caused. For example, severe acute respiratory syndrome, (SARS) and Middle east respiratory syndrome (MERSA) (1).

In the recent years a novel coronavirus caused a new pandemic that started from China in 2019 it was named as corona virus disease 2019 (COVID -19) which is a highly contagious viral illness. This catastrophic pandemic that was caused by severe acute respiratory syndrome coronavirus 2(SARS-CoV-2). COVID-19 had an enormous effect on the whole world resulting in more than 6 million deaths globally. The disease rapidly spread worldwide in late December 2019 at which the first case were detected in Wuhan, Hubei province, China (2). This urged the World Health Organization (WHO) to announce it as a global pandemic on March 11, 2020 (3).

Due to the high transmissibility of the virus countries worldwide were compelled to implement new policies –like social distances. This condition caused a huge impact on the healthcare since the new situation requires not only rapid vaccine development but also rapid testing. Due to the notable differences in the health care access economic response and findings the global response was unequal (4).

The early screening of colorectal cancer is crucial since it is according to GLOBOCAN 2020 data, CRC is the third most common and the second fatal cancer worldwide (5).

In 20% of the patients the disease can be presented as a metastatic CRC (mCRC). Additionally in about 50% of the patient the disease is localized then develops metastasis (6).

Many countries suspended the screening programs, particularly for colorectal cancer (CRC), subsequent to the world health organization’s announcement in March 2020 about the global pandemic of COVID -19 and the use of health care resources to control this pandemic (5).

The detection and treatment of colorectal cancer (CRC) have also been difficult during the COVID -19 pandemic. Hence any delay in the diagnosis and treatment of this cancer has irreversible consequences., as it is anticipated that a delay of 3 months and only a transition from stage I/T1 to stage II/T2 causes 88 additional deaths and it is imposed 12$ million higher expenses on health care systems (5). Additionally, a six-month delay in identification and treatment of colorectal cancer can result in additional 349 fatalities each month and $46million in addition to expanses over five-year period (7). Furthermore, it is predicted that patient survival will drop by more than 9% after a two - month delay in surgery and by 29% after 6-month delay (8).

Understanding how COVID -19 affects cancer diagnosis and therapy is crucial for health care systems in order to improve planning (5). As a result, the current study will investigate how the COVID 19 pandemic has affected colorectal cancer detection and treatment.

## 2 Problem statement

The COVID-19 pandemic, declared by the World Health Organization in March 2020, precipitated an unprecedented global health crisis that severely disrupted essential healthcare services, including those for colorectal cancer (CRC) the world’s third most prevalent and second deadliest cancer (3, 5). The reallocation of healthcare resources and the suspension of screening programs led to significant delays in the diagnosis and treatment of CRC (5). Given that timely intervention is critical for outcomes with predictions indicating that even a 3-month delay can lead to dozens of additional deaths and impose millions in healthcare costs (5, 7) it is imperative to systematically evaluate the full extent of the pandemic’s impact on CRC management. A comprehensive synthesis of the evidence is essential to quantify these disruptions, understand their consequences on disease stage and survival, and inform future healthcare policy to build more resilient cancer care systems.

## 3 Aim and objectives

This systematic review aims to assess the impact of the COVID-19 pandemic on management strategies, treatment outcomes, and overall survival rates of patients diagnosed with colorectal cancer. The specific objectives are to (1): evaluate changes in diagnostic pathways (2); analyze alterations in treatment modalities (surgery, chemotherapy, radiotherapy) (3); investigate the effects of care delays on disease progression and survival (4); identify challenges faced by healthcare systems; and (5) provide evidence-based recommendations for optimizing CRC management in future crises.

PICOS Element

1. Population: Adult patients (≥18 years) diagnosed with colorectal cancer
2. Intervention/Exposure: The COVID-19 pandemic (period from March 2020 onwards)
3. Comparator: Pre-pandemic period
4. Outcomes: Changes in diagnostic pathways, treatment modifications, stage at presentation, survival rates, and healthcare system challenges
5. Study Design: Retrospective cohort studies, prospective cohort studies, population-based analyses, modeling studies, cross-sectional surveys, and qualitative studies

## 4 Methodology

**Figure 1:**
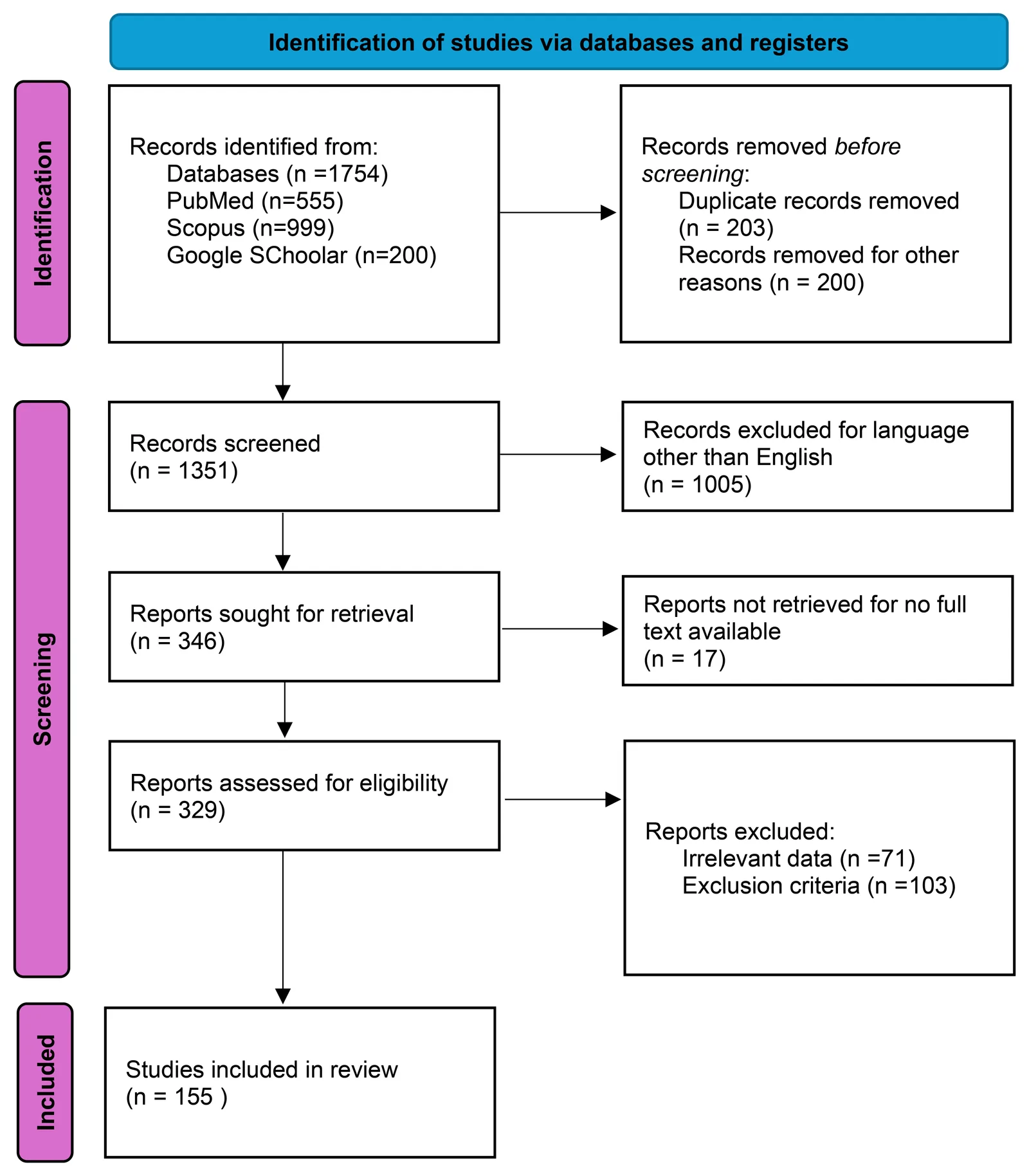
PRISMA flow diagram. PRISMA, Preferred Reporting Items for Systematic Reviews and Meta-Analyses.

This study was conducted as a systematic review following the PRISMA (Preferred Reporting Items for Systematic Reviews and Meta-Analyses) guidelines to ensure a comprehensive and transparent reporting process (**Figure 1**). The review protocol was registered with International Prospective Register of Systematic Reviews (PROSPERO; registration number CRD42025638100). This review did not require review or approval by the ethics committee as it used previously published data. The eligibility criteria were defined to include peer-reviewed studies published from 2020 onwards that focused on adult patients diagnosed with colorectal cancer and specifically addressed the impact of the COVID-19 pandemic on management strategies, treatment outcomes, or survival rates. Studies not published in English, those focusing on other cancer types or non-human subjects, abstracts without full text, publications with no extractable data, and duplicate studies were excluded.

A systematic search was performed using PubMed, Scopus and Google SChoolar database. The search strategy employed a combination of keywords and MeSH terms related to colorectal cancer (“colorectal cancer”, “colon cancer”, “rectal cancer”), COVID-19 (“COVID-19”, “SARS-CoV-2”), and outcomes (“treatment outcomes”, “management”, “survival”, “diagnosis”). An example search string was: (“colorectal cancer” OR “colon cancer” OR “rectal cancer”) AND (“COVID-19” OR “SARS-CoV-2”) AND (“treatment outcomes” OR “management” OR “survival” OR “diagnosis”). The study selection process involved a two-step screening approach: initially, titles and abstracts were screened for relevance, followed by a full-text review of potentially eligible studies against the inclusion criteria.

Data were extracted using a standardized form to collect information on study details (authors, publication year, country), patient demographics, changes in diagnostic pathways, alterations in treatment modalities, survival outcomes, and challenges faced by healthcare systems. The quality of the included observational studies was assessed using the Newcastle-Ottawa Scale. For data synthesis, a narrative approach was employed to summarize the findings according to key themes related to the review objectives, and subgroup analyses were planned to explore heterogeneity based on factors such as disease stage and treatment modality. Meta-analysis was not performed due to substantial heterogeneity in study designs, outcome measures, and reporting formats.

## 5 Results

### 5.1 Search results

### 5.2 Quality assessment

The methodological quality of the 155 included studies was assessed using the Newcastle-Ottawa Scale (NOS). Most studies (n=93, 60%) were rated as high quality, achieving scores of 8 or 9 out of 9. These studies were characterized by their use of large, often national or multicenter datasets, robust statistical analyses that adjusted for key confounders such as age and comorbidity, and clear comparisons between pre-pandemic and pandemic periods. A significant proportion of studies (n=56, 36%) were of moderate quality (NOS scores 5-7), typically due to single-center designs, smaller sample sizes, or a lack of long-term outcome data. Only six studies (4%) were deemed low quality, primarily due to a high risk of bias from methodological limitations such as the use of non-clinical proxy data for screening rates. The most common limitation across all studies, including high-quality ones, was their retrospective nature and the inability to account for all potential confounders, such as detailed socioeconomic factors or patient-level reasons for care avoidance. Despite this, the overall body of evidence was robust, with high-quality studies providing strong evidence on the impact of the pandemic on CRC screening, diagnosis, and treatment. (**Table 1**).

**Table 1:** Summary of methodological quality of included studies (assessed using the Newcastle-Ottawa Scale).

Quality rating	Number of studies	Percentage of studies	Common strengths	Common limitations
High Quality	93	~60%	- Large, population-based/national datasets - Prospective or robust multicenter design - Clear pre/post pandemic comparison - Adjustment for key confounders	- Retrospective design (common across all) - Lack of long-term survival/outcome data - Potential for unmeasured confounding
Moderate Quality	56	~36%	- Clearly defined cohorts and outcomes - Appropriate statistical methods - Focus on specific, relevant adaptations (e.g., FIT triage)	- Single-center design limiting generalizability - Smaller sample sizes - Short follow-up periods - Reliance on administrative/data coding accuracy
Low Quality	6	~4%	- Provided relevant, real-time data during the pandemic - Highlighted specific clinical scenarios	- Significant risk of bias (e.g., use of proxy data like search trends) - Lack of control group or adjustment for confounders - High potential for missing data

### 5.3 Study characteristics

The included studies comprised a heterogeneous mix of designs aimed at quantifying the pandemic’s impact from various angles. The vast majority were retrospective cohort studies utilizing hospital records, cancer registries, or administrative claims data to compare CRC metrics before and during the pandemic. These were complemented by population-based analyses from national or regional screening programs, providing a broad overview of screening participation and diagnostic delays. Modeling studies were employed to project the long-term consequences of care disruptions on cancer incidence and mortality. A smaller subset of studies included prospective cohort designs tracking service modifications in real-time, cross-sectional surveys assessing patient and provider experiences, and qualitative investigations exploring the psychosocial and behavioral impacts on patients (9–16). The evidence base was globally sourced, with significant contributions from North America, Europe, and Asia, reflecting the widespread nature of the disruptions.

### 5.4 Key findings

The synthesis of results reveals a cascade of negative consequences for colorectal cancer care, stemming from initial disruptions and leading to altered patient outcomes.

#### 5.4.1 Disruptions to CRC screening

The COVID-19 pandemic caused a severe global reduction in colorectal cancer (CRC) screening, evidenced by sharp declines in FIT testing (9-67%), colonoscopy volumes (up to 92%), and prolonged diagnostic wait times (10, 17–35). These disruptions led to a 13-36% drop in new CRC diagnoses, leaving thousands of cases undetected and causing a significant shift toward more advanced-stage disease at presentation (28, 29, 34). Modeling studies projected these delays would result in thousands of excess CRC cases and deaths in the long term (25, 32, 36, 37). The pandemic also worsened health disparities, with racial/ethnic minorities and socioeconomically disadvantaged groups experiencing steeper declines in screening access (37–40), while regions with robust, centralized screening programs were more resilient to disruptions (22).

#### 5.4.2 Diagnostic delays & advanced presentations

Diagnostic delays during the COVID-19 pandemic led to a significant shift toward more advanced colorectal cancer (CRC) at presentation (41–48). This was marked by a consistent increase in metastatic (Stage IV) disease, with proportions rising from 22% to 37% in some cohorts, alongside a decline in early-stage detections (46–49). Tumors were more locally advanced, with higher rates of T4 staging, nodal involvement, and angiolymphatic invasion (41, 42, 50). Clinically, this translated to more emergency presentations, such as obstructions and bleeding, and necessitated more complex treatments, including increased stoma formation and acute surgical resections (41, 43, 45). The collective evidence confirms that pandemic-related delays resulted in more severe disease at diagnosis, potentially worsening long-term survival.

#### 5.4.3 Adaptive treatment strategies with altered surgical volumes and shifts in oncological quality

The COVID-19 pandemic led to significant modifications in colorectal cancer treatment, including reduced elective surgical volumes (declines of 5.4% to over 45%) and a strategic shift toward neoadjuvant therapies, such as a 200% increase in short-course radiotherapy, to bridge delays (51–55). While short-term surgical safety was maintained with comparable complication and mortality rates (56, 57), concerning indicators of more advanced disease were observed, including higher rates of lymphovascular invasion, stoma formation, and a reduction in minimally invasive surgeries (9, 58, 59). These changes suggest that despite adaptive strategies to preserve immediate patient safety, the pandemic likely resulted in treating more complex cases, with potential implications for long-term oncological and functional outcomes.

#### 5.4.4 Healthcare system adaptations

Healthcare systems rapidly implemented adaptive strategies to sustain colorectal cancer care during the COVID-19 pandemic (11, 60–64). Key innovations included the widespread adoption of FIT-based triage to prioritize endoscopy for high-risk patients, the establishment of COVID-free “cold sites” to protect surgical capacity, and the rapid integration of telemedicine for consultations and multidisciplinary meetings, with 94% of practices in one study adopting remote care (11, 60, 61). These measures, alongside accelerated treatment pathways for urgent cases, enabled systems to maintain critical services and effectively control infection risk among vulnerable cancer patients, demonstrating a pivotal shift toward more efficient and resilient care delivery.

#### 5.4.5 Patient outcomes & survival impact

The pandemic negatively impacted colorectal cancer (CRC) survival through two primary pathways: direct SARS-CoV-2 co-infection and indirect treatment delays (65–69). A large Japanese registry found patients diagnosed during the pandemic had a 25% higher mortality risk (69), while a French study reported a dramatically worse one-year survival of 76% for CRC patients with COVID-19 versus 93% without (1699). Postoperative COVID-19 infection was particularly lethal, with one study reporting a 60% mortality rate (1808). Modeling studies projected that diagnostic and therapeutic delays would lead to hundreds of excess CRC deaths in the long term (415). However, some resilient healthcare systems, such as in Taiwan, showed that adaptive strategies could mitigate these adverse outcomes, demonstrating no significant difference in one-year mortality (30). The evidence confirms a dual burden of direct mortality from co-infection and a potential long-term survival disadvantage from care disruptions.6. Psychosocial & Behavioral Impacts.

#### 5.4.6 Profound psychosocial distress and behavioral changes exacerbated cancer experience

The pandemic significantly worsened the psychological well-being and health behaviors of colorectal cancer patients and survivors. Qualitative research revealed heightened anxiety about COVID-19 vulnerability, intense social isolation from severed support networks, and detrimental lifestyle changes, including increased comfort eating and alcohol use (70). This fear directly impacted healthcare engagement, as shown by a dramatic rise in “no-show” rates for urgent endoscopies—from 15.1% to 48.2%—as patients weighed the risk of infection against their cancer symptoms (71). This avoidance behavior likely contributed to the observed trend of patients presenting with more advanced disease and complex comorbidities during the pandemic highlighting the critical interplay between mental health, behavior, and clinical outcomes (72, 73).

#### 5.4.7 Increased vulnerability: cancer patients faced higher risks of severe COVID-19 outcomes

Cancer patients, including those with colorectal cancer, were a high-risk group for severe COVID-19. Retrospective studies reported substantial rates of hospitalization (40%), severe respiratory illness (20%), and 30-day mortality (12%) in this population (74). While advanced age and immune checkpoint inhibitors were linked to worse outcomes, recent chemotherapy or metastatic disease were not consistent risk factors, highlighting a complex risk profile (75). A validated comorbidity score was developed to predict severe outcomes in cancer patients (AUC 0.82-0.85), aiding risk stratification (76). Critically, continuation of most systemic anti-tumor therapies did not significantly increase infection rates, though palliative intent treatment was associated with higher hospitalization, underscoring the need to carefully balance infection risk with ongoing cancer care (77).

#### 5.4.8 Technological adaptations

The pandemic accelerated the adoption of digital tools to manage clinical pathways. One center rapidly deployed a customized, Excel-based data management system with VBA programming to handle patient referrals, enabling robust, real-time support for a FIT-based triage pathway. This low-cost, agile solution demonstrated that effective digital infrastructure could be quickly established to ensure the safe prioritization of colorectal cancer diagnostics during a crisis (78).

#### 5.4.9 Adverse lifestyle shifts

For individuals living with and beyond cancer, the pandemic led to significant negative changes in health behaviors, particularly a decline in fruit and vegetable intake and an increase in snacking. These detrimental dietary changes disproportionately affected survivors who were women, those with lower educational attainment, individuals who were shielding, and those with poor sleep or limited social contact, highlighting exacerbated health inequalities and the need for targeted nutritional support (79).

#### 5.4.10 Digital divide

The shift to digital health revealed significant disparities in digital literacy among cancer survivors, which was strongly associated with older age, lower income, and lower education. This divide had tangible consequences: survivors with lower digital literacy were less likely to use online health resources, while higher digital literacy predicted greater adoption of mobile apps and eHealth engagement. These findings underscore the need to address the digital divide to ensure equitable access to cancer care (80).

## 6 Discussion

This systematic review confirms that the COVID-19 pandemic, declared by the WHO in March 2020 (3), had a profound and detrimental impact on the entire continuum of colorectal cancer (CRC) care, validating early predictions of severe consequences from care delays (5, 8) The findings demonstrate a catastrophic cascade, beginning with the widespread suspension of screening programs (5), which led to global reductions in screening participation by 30-80% and drastic declines in colonoscopy volumes (30). This initial disruption directly caused the anticipated “stage shift,” with a significant increase in advanced-stage and metastatic diagnoses at presentation (46, 48, 49) and a higher frequency of emergency surgeries (43, 45). The tangible manifestation of these delays was evidenced by a significantly higher mortality risk for patients diagnosed during the pandemic (81), tragically illustrating the irreparable consequences forecasted at the pandemic’s onset (10, 17–35).

This review highlight that the pandemic’s negative impact was not uniform across all patients, but rather more severe for those with more advanced initial disease and early-stage patients may have been more vulnerable to delays because they relied on screening programs that were suspended. A key finding of this review is that the initial TNM stage at diagnosis influenced the impact of the COVID-19 pandemic on colorectal cancer outcomes. Patients presenting with advanced-stage disease (Stage III–IV) experienced disproportionately worse outcomes, including higher rates of emergency presentation, stoma formation, and urgent surgical resection (41, 43, 45). Advanced tumors are biologically more aggressive and less tolerant of treatment delays, making these patients particularly vulnerable during periods of healthcare disruption (8). In contrast, early-stage cancers (Stage I–II), which are commonly detected through routine screening, were more affected by the suspension of screening programs, contributing to fewer early-stage diagnoses and an increase in metastatic presentations during the pandemic (5, 46–49). Although one included study suggested that treatment delays of up to six weeks did not adversely affect disease-free survival in Stage I–III disease (13), the long-term consequences of pandemic-related stage migration remain uncertain. These findings highlight the importance of stage-specific prioritization strategies during future healthcare crises to minimize delays and optimize outcomes across all disease stages.

Despite these severe disruptions, the global crisis also catalyzed rapid and innovative adaptations within healthcare systems, showcasing a degree of resilience. The implementation of triage protocols, such as FIT-based prioritization, and the establishment of COVID-free “cold sites” were critical strategies that helped maintain essential cancer services for high-risk patients (11, 61). Furthermore, the shift toward neoadjuvant therapies and the integration of telemedicine allowed for continuity of care while mitigating infection risks (51, 60). However, this review also highlights that the pandemic’s burden was not borne equally, exacerbating pre-existing health disparities. Vulnerable populations, including racial/ethnic minorities and those of lower socioeconomic status, experienced steeper declines in screening and greater barriers to care, reflecting the “uneven global response” noted in the introduction (38, 40).

In conclusion, the findings from this synthesis underscore the critical vulnerability of cancer care systems to global public health emergencies. The pandemic inflicted a dual burden on CRC patients: indirect harm through diagnostic and treatment delays leading to more advanced disease, and direct harm from the lethal synergy of COVID-19 co-infection (67, 68). The long-term projections of excess CRC deaths serve as a stark warning of the lingering consequences (32, 82). Moving forward, the lessons learned from both the disruptions and the successful adaptations such as resilient screening infrastructure and risk-stratified care pathways must be formalized into future preparedness plans to protect cancer patients during subsequent crises.

## Data Availability

The original contributions presented in the study are included in the article/supplementary material. Further inquiries can be directed to the corresponding author.
